# Differences between persons with and without disability in HIV prevalence, testing, treatment, and care cascade in Tanzania: a cross-sectional study using population-based data

**DOI:** 10.1186/s12889-023-17013-8

**Published:** 2023-10-25

**Authors:** David Chipanta, Sophie Mitra, Silas Amo-Agyei, Minerva Rivas Velarde, Kofi Amekudzi, Connie Osborne, Janne Estill, Olivia Keiser

**Affiliations:** 1 University of Geneva, Geneva, Switzerland; 2 Joint United Nations Programme on HIV/AIDS (UNAIDS), Geneva, Switzerland; 3 Fordham University, New York, USA; 4 University of Lausanne, Lausanne, Switzerland; 5 Geneva School of Health Science, Geneva, Switzerland; 6 International Labour Organisation, Geneva, Switzerland; 7 National HIV/AIDS/STI/TB Council, Lusaka, Zambia

**Keywords:** Persons with disability, HIV testing, care cascade, UNAIDS 90-90-90 target, 95-95-95 target, Leave no one behind, Disability-inclusive-policies

## Abstract

**Background:**

Persons with disability may have a higher HIV prevalence and be less likely than persons without disability to know their HIV-positive status, access antiretroviral therapy (ART), and suppress their HIV viral load (HIV care cascade). However, studies examining differences between persons with and without disability in HIV prevalence and the HIV care cascade are lacking. Using the Tanzania HIV Impact Survey (THIS) data collected between October 2016 and August 2017, we assessed differences in HIV prevalence and progress towards achieving the 2020 HIV care cascade target between persons with and without disability.

**Methods:**

Using the Washington Group Short Set (WG-SS) Questions on Disability, we defined disability as having a functional difficulty in any of the six life domains (seeing, hearing, walking/climbing, remembering/ concentrating, self-care, and communicating). We classified respondents as disabled if they responded having either “Some Difficulty”, “A lot of difficulties” or “Unable to” in any of the WG-SS Questions. We presented the sample characteristics by disability status and analyzed the achievement of the cascade target by disability status, and sex. We used multivariable logistic regressions, and adjusted for age, sex, rural-urban residence, education, and wealth quintile.

**Results:**

A total of 31,579 respondents aged 15 years and older had HIV test results. Of these 1,831 tested HIV-positive, corresponding to an estimated HIV prevalence of 4.9% (CI: 4.5 — 5.2%) among the adult population in Tanzania. The median age of respondents who tested HIV-positive was 32 years (with IQR of 21—45 years). HIV prevalence was higher (5.7%, 95% CI: 5.3—7.4%) among persons with disability than persons without disability (4.3%, 95% CI: 4.0 — 4.6%). Before adjustment, compared to women without disability, more women with disability were aware of their HIV-positive status (n = 101, 79.0%, 95% CI: 68.0—87.0% versus n = 703, 63.0%, 95% CI: 59.1—66.7%) and accessed ART more frequently (n = 98, 98.7%, 95% CI: 95.3—99.7% versus n = 661, 94.7%, 95% CI: 92.6—96.3%). After adjusting for socio-demographic characteristics, the odds of having HIV and of accessing ART did not differ between persons with and without disability. However, PLHIV with disability had higher odds of being aware of their HIV-positive status (aOR 1.69, 95% 1.05—2.71) than PLHIV without disability. Men living with HIV and with disability had lower odds (aOR = 0.23, 95% CI: 0.06—0.86) to suppress HIV viral loads than their counterparts without disability.

**Conclusion:**

We found no significant differences in the odds of having HIV and of accessing ART between persons with and without disability in Tanzania. While PLHIV and disability, were often aware of their HIV-positive status than their non-disabled counterparts, men living with HIV and with disability may have been disadvantaged in having suppressed HIV viral loads. These differences are correctable with disability-inclusive HIV programming. HIV surveys around the world should include questions on disability to measure potential differences in HIV prevalence and in attaining the 2025 HIV care cascade target between persons with and without disability.

**Supplementary Information (Note for correction: please replace the titles of the supplimentary tables as indicated in the section for supplimintary form):**

The online version contains supplementary material available at 10.1186/s12889-023-17013-8.

## Introduction

Persons with disability are disadvantaged in several domains of life [1–6]. Globally, regardless of measurement methods used, persons with disability have lower educational attainment, employment, food security, and wealth. They also suffer higher exposure to economic shocks, health expenditures and multidimensional poverty than persons without disability [1–3]. They comprise 15% of the global population, equivalent to one billion people [2]. Most persons with disability live in lower- and middle-income countries, where they face multiple constraints [2]. The Convention on the Rights of Persons with Disabilities (CRPD) provides a set of legally binding commitments to promote and protect the full and equal enjoyment of human rights and freedoms for persons with disability [7]. The CRPD defines persons with disability to include persons with long-term physical, mental, intellectual, or sensory impairments, which when interacting with various barriers may hinder their full and effective participation in society on an equal basis with others [7]. This article uses the term “disability” rather than “disabilities” to reflect disability as a universal experience and not an intrinsic characteristic of the person. Unfortunately persons with disability are neglected in accessing life-saving HIV services [4–6] despite the governments, globally, making significant progress in achieving the UNAIDS 90—90—90 target by 2020 with the ultimate aim to end the AIDS epidemic by 2030 [8]. The 90—90—90 target sought to ensure that by 2020, 90% of people living with HIV (PLHIV) would know their HIV-positive status, 90% of those knowing their status would have accessed anti-retroviral therapy (ART), and 90% of PLHIV accessing ART would have suppressed their HIV viral loads protecting their health and limiting the spread of HIV [9]. The remarkable progress made in achieving the 90—90—90 target encouraged United Nations (UN) member states and other stakeholders to increase their ambition, approving the 95—95—95 target, seeking to meet it by 2025 across all PLHIV, within all demographic groups, and geographic settings [10].

Studies show that persons with disability, especially women, are disproportionally affected by HIV infection while being less likely to access HIV prevention and treatment services than persons without disabilities [5, 6, 11–17]. These studies have shown that persons with disability often face delays and barriers in accessing HIV testing, ART and care, ranging from long distances to HIV services, negative attitudes from society and health providers, to high costs [5, 6, 11–18]. These barriers include poor governance, leadership, financing, and lack of appropriate data [5, 6, 11, 16, 19]. However, these studies and others have also shown that sometimes persons with disability have accessed HIV testing, treatment, and care as much as persons without disability, depending on the type of disability and functional difficulties [5, 6, 11–18]. For example, a study in South Africa found the prevalence of HIV infection was similar among persons with and without disability — 16.7% versus 16.2%. However, the prevalence was elevated among persons with visual, hearing or speech disabilities [15]. Regarding the 90—90—90 target, the study in South Africa examined access to ART among persons with and without disability, finding that ART exposure was higher among PLHIV with disability than PLHIV without disability [15]. In contrast, in Tanzania, disabled caregivers living with HIV of orphans and vulnerable children were 42% less likely to have access to ART than non-disabled caregivers [20]. None of these studies analysed the difference between persons with and without disability across the entire 90—90—90 target. Such studies are necessary to provide evidence on whether persons with disability are left behind in accessing life-saving HIV care cascade services. Using the population-based HIV impact assessment (PHIA) survey data for Tanzania, also known as the Tanzania HIV population-based Impact Survey (THIS), collected in 2016—2017, we aimed to assess the differences in HIV prevalence and attainment of the 90—90—90 target between persons with and without disability. We hypothesized that persons with disability, compared to persons without disability, have a higher HIV prevalence, but lower rates of knowing their HIV-positive status, accessing ART, and suppressing their HIV viral load.

## Materials and methods

The THIS 2016–2017 is a nationally representative, cross-sectional survey of households across Tanzania. The Government of Tanzania led the data collection and analysis of the survey data through the Tanzania Commission for AIDS (TACAIDS) and Zanzibar AIDS Commission (ZAC), and several government agencies with funding from the U.S. President’s Emergency Plan for AIDS Relief (PEPFAR) and technical assistance through the U.S. Centers for Disease Control and Prevention (CDC) and ICAP at Columbia University. Before data collection, the THIS protocol was reviewed and approved by the institutional review boards of CDC, Columbia University, Westat, the National Institute for Medical Research, and the Zanzibar Medical Research and Ethics Committee [21].

THIS collected a range of health and socio-demographic data to evaluate the impact of HIV programs funded by PEPFAR in Tanzania. After field workers obtained informed consent, they administered a household questionnaire to the head-of-household in participating households, who provided information on the household relating to wealth and other socio-demographic characteristics. Then, the individual questionnaire was administered to eligible and consenting household members The survey also included biomarkers for example confirming HIV-positive status, access to ART and HIV viral load suppression with laboratory confirmed tests according to national testing algorithms. Each interview was administered using tools that have been validated and internationally used in PEPFAR supported countries, described in the THIS report [21]. The personal interviews assessed a range of HIV-related variables, including rapid HIV testing, and access to HIV treatment.

THIS comprised the household, adult, adolescent, and biomarker datasets derived from respective questionnaires. Questionnaire and biomarker data were collected on mobile tablet computers using an Open Data Kit software application. The questionnaires were prepared in English, translated into Kiswahili, and translated back into English by a different person to assess the accuracy of the translation [21]. We used the household, adult, and biomarker data sets. A detailed description of the THIS datasets, survey design, sampling, variables, and analytic guidance are described and available at the PHIA Project website at https://phia-data.icap.columbia.edu/files [21]We estimated the HIV prevalence and its 95% confidence interval (CI) and restricted our analysis to adults ≥ 15 years of age with a confirmed HIV-positive status.

### Outcomes and variable description

The primary outcomes were HIV prevalence (the percentage of people who had tested HIV-positive), the percentage of PLHIV who were aware of their HIV-positive status, percentage of PLHIV aware of their HIV-positive status who had accessed ART, and percentage of PLHIV on ART who had suppressed HIV viral load. The primary predictor was disability as measured by the Washington Group Short Set Questions (WG-SS) on disability. The WG-SS has been extensively tested and validated in all regions of the world [19] and it has been proven to reliably measure the prevalence, type, and severity of disability or functional limitations [22]. The WG-SS used are: “Do you have difficulty seeing, even if wearing glasses?”; (ii) “Do you have difficulty hearing, even if using a hearing aid?”; (iii) “Do you have difficulty walking or climbing steps?”; (iv) “Do you have difficulty remembering or concentrating?”; (v) “Do you have difficulty with self-care such as washing all over or dressing?”; and (vi) “Using your usual (customary) language, do you have difficulty communicating, for example understanding or being understood?.” The functional difficulties by domain type were classified by the following levels —1 – “No difficulty”, 2 – “Some difficulty”, 3 – “A lot of difficulties”, 4 – “Unable to”, 5 – “Not applicable”, and 8 – “Don’t know”. We coded “Not applicable” and “Don’t know” as missing and combined “Some Difficulty”, “A lot of difficulties” and “Unable to”, to reflect any difficulty. We label this variable as disability, although disability is a broad term that cover other difficulties such as psychosocial ones that are not captured by the WG-SS. We excluded albinism because albinism has not been measured in internationally comparable questions [23]. Other variables identified through a literature review associated with the 90–90–90 target included sex, age, rural-urban-residence, education and wealth levels [5, 6, 11, 13–18, 24].

We used the variables provided in THIS data set and described in the THIS 2016–2017 adult, biomarker and household codebooks, coding HIV status negative or positive, undefined HIV status as missing and excluded from the analysis. Awareness of HIV-positive status was defined as having both a laboratory confirmed HIV-positive test result and self-reporting HIV result positive. Accessing ART reflected both self-reported using ART and laboratory-confirmed ART use. HIV RNA viral load less than 1,000 copies per ml of plasma denoted viral load suppression. Sex was self-reported, i.e., male or female. Age was self-reported and categorized 15–24, 25–34, 35–44, 45–54, and 55+. Rural-urban residency reflected self-reported residence type. From the education variable, we categorized education as no education, primary education and secondary and higher. We combined pre-primary and primary education into primary education, and post-primary education, secondary and higher education, and university into secondary and higher education due to lack of sufficient observations in each category for analysis [21]. The wealth quintiles assessed household wealth, ranking households based on household characteristics such as construction materials for walls, floors and roof of the household dwelling, source of water, availability of electricity and type of sanitation facilities, and asset ownership, from wealth quintile 1 (Q1) representing the poorest households to Q5 the wealthiest. Variables for assets are analyzed using the Principal Component Analysis, a statistical technique transforming several correlated variables into uncorrelated components. The first component of the model is used as a summary indicator for wealth (the wealth index) as recommended by the Demographic and Health Survey method. Households are then classified into quintiles using the wealth index. In general, households in higher wealth quintiles should be wealthier than those in lower quintiles [25–27].

We included respondents who had a confirmed HIV test result (negative or positive) for assessing HIV prevalence. For HIV care cascade, we included adults who had an HIV-positive test result, then among these, adults who were aware of their HIV-positive status. Among adults who were aware of their HIV-positive status we captured adults who were accessing ART and among those who accessed ART, those suppressing their HIV viral load.

### Analysis

We analyzed differences between persons with and without disability in attaining the 90—90—90 target in three steps. In step one, we presented the estimates of sample characteristics, and their 95% CI, based on disability status. In step two, we examined the differences in the 90-90-90 target by sex and tested for differences using the Pearson Chi-squared design-based test. In step three, we examined the differences between persons with and without disability using multivariable logistic regressions in the HIV care cascade, adjusting for age, sex, rural-urban-residence, education, and wealth quintile. Because several studies have examined the factors associated with the HIV care cascade, we present only the odds ratios for the disability variable of the unadjusted and adjusted models [28, 29, 24]. In Supplementary Tables A1, B1 and C1, we show the odds ratios for the disability variable and the covariates for unadjusted and adjusted models. We used survey weights to account for non-response using chi-squared automatic interaction detector (CHAID) analysis, non-coverage, and probability selection; 95% CI were estimated using jackknife replicate weights. We disaggregated the analyses by sex. Missing data were less than 2% of the sample and were excluded from the analyses. Stata version 14.2 was used for analyses.

## Results

**Fig. 1:**
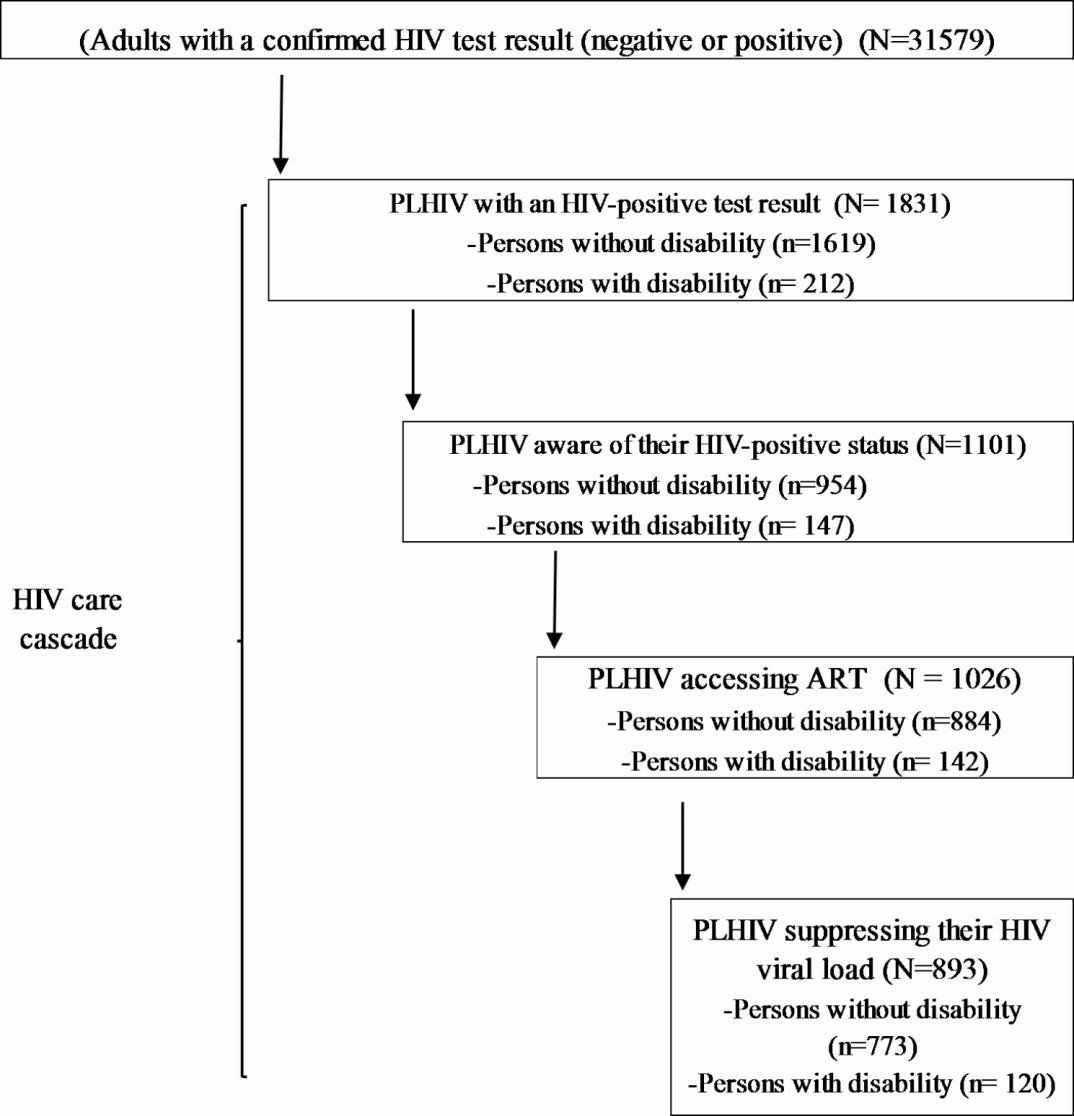
Flowchart for data extraction

A total of 31,579 respondents aged 15 years and older had HIV test results. Of these 1,831 (representing 4.9%, CI: 4.5 — 5.2%) were HIV-positive. Of the 1,831 PLHIV, 1101 (60.6%, 57.1 − 63.9%) were aware of their HIV-positive status, of whom 1026 (93.6%, 91.7 − 95.2%) accessed ART, and 893 (87.0%, 84.1 − 89.5%) of the 1026 had suppressed HIV viral load (Fig. 1).

Table 1 shows the sample descriptive statistics. HIV prevalence was 4.9% (N = 1831, 95% CI: 4.5 — 5.2%) among adults 15 years or older in Tanzania. The median age of PLHIV was 32 (interquartile range of 21–45 years). Nearly two-thirds were women, they had a primary school education level, and were from households in wealth quintiles 3—5 (Table 1).

**Table 1:** HIV prevalence, sample characteristics among PLHIV and the 90-90-90 target by disability status, THIS 2016–2017

Variable	Not disabled		Disabled	p-value	Total	
HIV status						
HIV-positive	1619 (4.3, 4.0–4.6)		212 (5.7, 5.3–7.4)	0.002	1831 (4.9, 4.5–5.2)	
HIV-negative	26,826 (86.6, 86.0–87.1)		2922 (8.6, 8.1–9.1)	0.002	29,748 (95.1, 94.8–95.5)	
**Total**	28,445 (90.8, 90.4–91.3)		3134 (9.2, 8.7–9.7)		31,579 (100)	
**Sample descriptive statistics of PLHIV by disability status (n = 1831)**						
**Disability status**	1619 (88.2, 86.4–89.8)		212 (11.8, 10.2–13.6)		1831 (100.0)	
**Gender**						
Male	489 (33.8, 30.9–36.8)		75 (34.3, 25.5–41.9)	0.0204	564 (33.8, 31.3–36.5)	
Female	1130 (66.2, 63.2–69.1)		137 (65.7, 58.1–72.5)	0.8876	1267 (66.2, 63.5–68.7)	
**Age (years)**						
15–24	168 (11.1, 9.3–13.2)		3 (1.8, 0.5–5.9)		171 (10.0, 8.4–11.9)	
25–34	466 (28.8, 25.8–32.0)		14 (6.7, 2.2–3.4)		480 (26.2, 23.3–29.3)	
35–44	540 (32.7, 29.8–35.8)		62 (29.4, 22.2–37.9)		602 (32.3, 29.6–35.1)	
45–54	285 (18.2, 15.9–20.7)		64 (28.5, 20.9–37.4)		349 (19.4, 17.3–21.7)	
55+	160 (9.2, 7.5–11.2)		69 (33.6, 27.2–40.8)	< 0.001	229 (12.1, 10.3–14.1)	
**Residence**						
Rural	959 (54.4, 49.8–59.0)		125 (54.8, 44.6–64.6)		1084 (54.5, 49.7–59.1)	
Urban	660 (40.2, 36.1–44.4)		87 (45.2, 35.4–55.4)	0.9422	747 (45.5, 40.9–50.3)	
**Education**						
No Education	328 (17.0, 14.8–19.4)		39 (2.1, 1.4–3.1)		367 (19.0, 16.7–21.6)	
Primary	1100 (58.7, 55.6–61.8)		153 (8.5, 7.3–10.0)		1253 (67.2, 64.2–70.2)	
Secondary & higher	189 (12.6, 10.5–14.9)		20 (1.2, 0.6–2.1)		209 (13.7, 11.7–16.0)	
Missing	2		0		2	
**Wealth Quintile**						
Q1 – poorest	295 (16.4, 13.6–19.7)		36 (13.9, 8.3–22.3)		331 (16.1, 13.4–19.3)	
Q2	300 (17.8, 15.3–20.8)		50 (23.0, 15.9–32.1)		350 (18.5, 16.0–21.2)	
Q3	445 (24.9, 21.6–28.5)		56 (27.6, 20.2–36.4)		501 (25.2, 21.9–28.8)	
Q4	349 (23.3, 20.3–26.7)		41 (21.7, 14.9–30.3)		390 (23.1, 20.2–26.3)	
Q5 – Richest	230 (17.5, 14.5–21.0)		29 (13.9, 9.5–19.8)		259 (17.1, 14.3–20.3)	
**90-90-90 target by HIV status**						
Awareness of HIV-positive status	954 (58.8, 55.1–62.3)		147 (73.9, 65.0–81.2)	0.003	1101 (60.6, 57.1–63.9)	
Access to ART	884 (93.0, 90.8–94.7)		142 (97.4, 92.7–99.1)	0.055	1026 (93.6, 91.7–95.2)	
HIV viral load suppression	773 (87.8, 84.6–90.3)		120 (83.1, 74.0–89.5)	0.212	893 (87.0, 84.1–89.5)	

Design-based Pearson Chi-squared test. 45 (2,8% of 1,619) observations were missing in the variable for the access to ART for people without disability and 4 (1,9% of 212) observations for persons with disability. HIV prevalence was based on respondents with an HIV test result (n = 31,579). PLHIV (n = 1831); PLHIV without disability (n = 1619) and PLHIV with disability (n = 212).

Approximately, 11.8% (n = 212, 95% CI: 10.2—13.6%) of PLHIV had a disability. The most common functional difficulties were related to seeing, walking, and hearing—difficulties in communicating or self-care were least reported (Supplementary Table 2). More women living with HIV than men living with HIV were disabled but the differences were not statistically significant (Table 1).

Respondents with disability had a higher HIV prevalence (n = 212, 5.7%, 95% CI: 5.3—7.4%) than respondents without disability (n = 1,619, 4.3%, 95% CI: 4.0—4.6%). Awareness of HIV-positive status and access to ART were higher among PLHIV with disability than PLHIV without disability (Table 1).

**Fig. 2:**
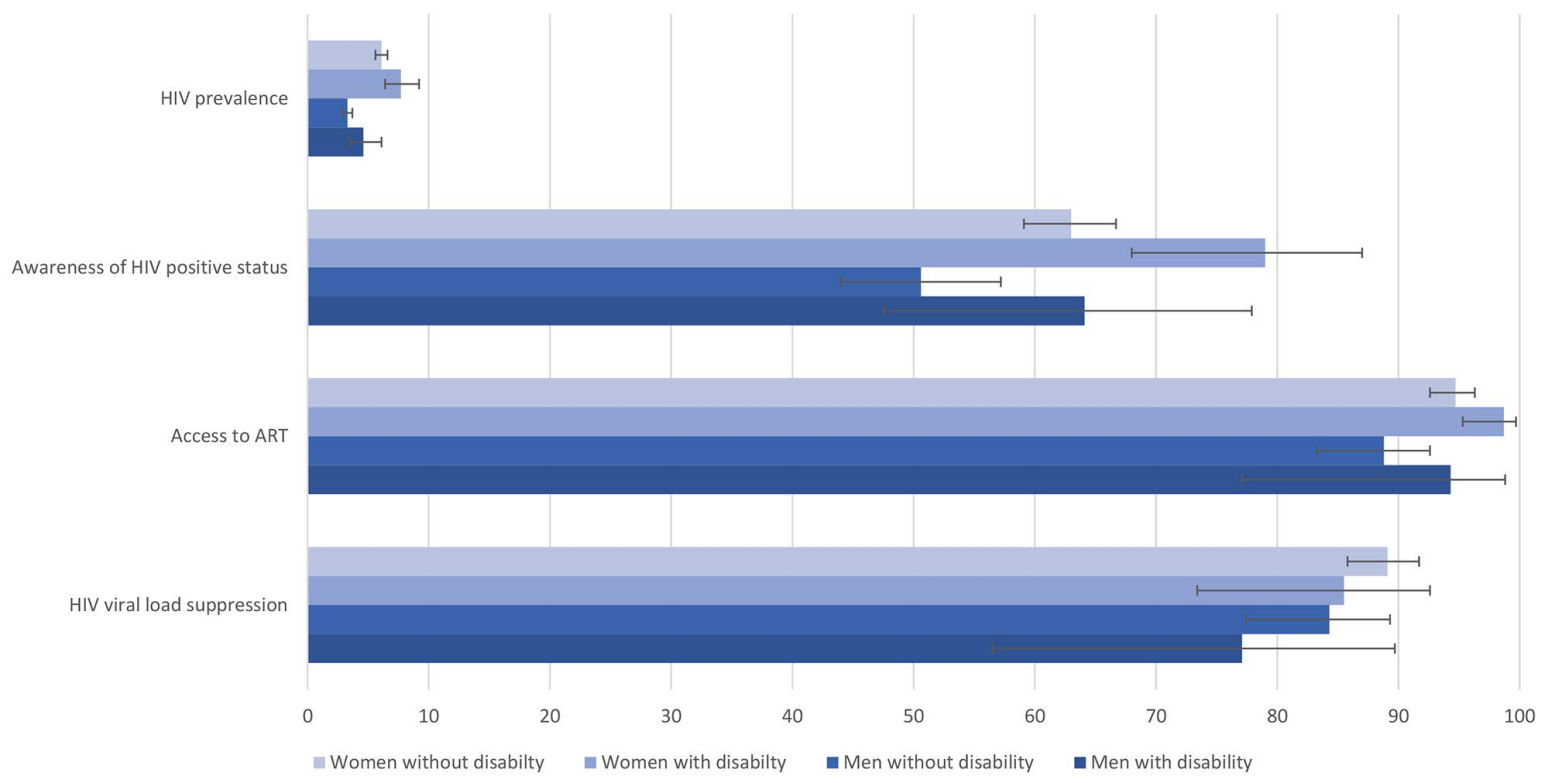
The proportions of people who had tested HIV-positive, had known their HIV-positive status, had accessed ART, and had suppressed HIV viral load by disability status and sex (Point estimates and 95% CI)

Figure 2 shows the crude proportions of men and women who had tested HIV-positive, knew their HIV-positive status, accessed ART and suppressed HIV viral load by disability status. The HIV prevalence was higher among women (n = 137, 7.7%, 95% CI: 6.4%—9,2%) and men (n = 75, 4.6%, 95% CI 3.5%—6.1%) with disability than among women (n = 1130, 6.1%, 95% CI: 5.6—6.6%) and men (n = 489, 3.3%, 95% CI: 2.9—3.7%) without disability. Women living with HIV and with disability were more frequently aware of their HIV-positive status (n = 101, 79.0%, 95% CI: 68.0—87.0%) versus (n = 703, 63.0%, 95% CI: 59.1—66.7%) and accessed ART (n = 98, 98.7%, 95% CI: 95.3—99.7% versus n = 661, 94.7%, 95% CI: 92.6—96.3%) than women living with HIV and without disability. Compared to men without disability, men with disability were more frequently HIV-positive, aware of their HIV-positive status, and had accessed ART, but less frequently suppressed HIV viral load, although the 95% confidence intervals overlapped. In general, women with or without disability had higher HIV prevalence, awareness of HIV prevalence, access to ART and HIV viral load suppression than their male counterparts (Fig. 2).

Table 2 presents the adjusted odds ratios (aOR) for HIV prevalence and attaining the 90—90—90 targets, disaggregated by sex. Neither HIV prevalence nor access to ART were statistically different between PLHIV with disability and PLHIV without disability after adjusting for age, sex, rural-urban-residence, education, and wealth quintile. The same results were seen in the analyses restricted to women or men (Table 2). PLHIV with disability had higher adjusted odds (aOR 1.69, 95% 1.05—2.71) of being aware of their HIV-positive status than their peers without disability. Women with disability had higher odds of being aware of their HIV-positive status than their peers without disability; in contrast this was not observed in men. However, men living with HIV with disability had lower odds (aOR = 0.23, 95% CI: 0.06—0.86) of suppressing their HIV viral load than men without disability (Table 2).

**Table 2:** The odds of having HIV, awareness of HIV-positive status, access to ART and HIV viral load suppression among persons with and without disability disaggregated by sex

Variable	HIV-status	p-value		Awareness of HIV-positive status	p-value		Accessing ART	p-value		HIV viral load suppression	p-value
Women and men (Unadjusted)											
Not disabled(ref)	1			1			1			1	
Disabled	1.35 (1.13–1.60)	0.002		1.99 (1.28–3.09)	0.004		2.84 (0.77–10.41)	0.111		0.69 (0.38–1.25)	0.209
**N**	31,579			1782			1101			1026	
**Women and men (Adjusted)§**											
Not disabled(ref)	1			1			1			1	
Disabled	1.03 (0.86–1.23)	0.768		1.69 (1.06–2.68)	0.029		1.56 (0.45–5.46)	0.473		0.55 (0.26–1.18)	0.120
**N**	31,552			1780			1100			1025	
**Women (Unadjusted)**											
Not disabled(ref)	1			1			1			1	
Disabled	1.27 (1.05–1.55)	0.018		2.21 (1.20–4.05)	0.012		4.27 (0.97–18.73)	0.054		0.72 (0.33–1.58)	0.397
**N**	17,829			1,230			804			759	
**Women (Adjusted)**											
Not disabled(ref)	1			1			1			1	
Disabled	1.06 (0.86–1.31)	0.585		1.93 (0.99–3.76)	0.053		2.08 (0.41–10.62)	0.364		0.71 (0.29–1.72)	0.431
**N**	17,812			1228			803			758	
**Men (unadjusted)**											
Not disabled(ref)	1			1			1			1	
Disabled	1.43 (1.03–2.00)	0.034		1.74 (0.82–3.68)	0.140		2.08 (0.23–19.08)	0.501		0.63 (0.22–1.79)	0.371
**N**	13,750			552			297				
**Men (Adjusted)**											
Not disabled(ref)	1			1			1			1	
Disabled	0.98 (0.70–1.38)	0.926		1.30 (0.58–2.91)	0.514		1.43 (0.16–12.59)	0.739		0.23 (0.06–0.81)	0.024
**N**	13,740			552			297			267	

Unadjusted and adjusted odds ratios, 95% confidence intervals, p-values. Reference = no disability. P-values are from wald tests of logistic regressions. Models were adjusted for age, rural-urban residence, education, and wealth quintile. § The model was adjusted for sex in addition to age, rural-urban-residence, education, and wealth quintile.

We tested for the interaction between disability and sex in the regression for HIV viral load suppression to understand whether the association between HIV viral load suppression and disability was different between men and women. The adjusted odds ratio for the female sex and disability interaction term was 1.03, suggesting that the association between disability and HIV viral load suppression did not differ between women and men.

## Discussion

This study aimed to assess, for Tanzania, the differences in HIV prevalence, awareness of HIV-positive status, access to ART, and HIV viral load suppression between persons without and with disability. We found no significant difference in HIV prevalence between persons without and with disability after adjusting for covariates. However, PLHIV with disability, in particular women, were more likely to be aware of their HIV-positive status than peers without disability; this finding was not observed in men. Men living with HIV with disability on ART were less likely to have suppressed their HIV viral load than their counterparts without disability. These results have policy implications for disability-inclusive interventions needed to achieve the 95-95-95 target.

Our first result contradicts studies that found that persons with disability have a higher risk of HIV infection than the general population; and that HIV prevalence was often twice higher among women with disability than women without disability, with no differences between men with and without disability [5, 6, 11–17]. Further, disability is more concentrated among older than younger persons, women than men, rural than urban dwellers, less educated than more educated, and poor than wealthy households, consistent with the results of our study [1–3, 30]. In our study, HIV prevalence was higher among persons with disability before adjusting for age, sex, rural-urban-residence, education, and wealth quintile. After adjusting for these covariates, HIV prevalence was no longer different between persons with and without disability. This result suggests that the higher crude HIV prevalence among persons with disability is caused by co-factors and their associations with disability. The HIV prevention, treatment cascade services delivered to persons with disability that consider the effect of these co-factors may help reduce the burden of HIV among persons with disability.

Our second result showed that persons with disability, in particular women, were more often aware of their HIV-positive status than persons without disability. Access to ART did not differ between persons with and without disability. Studies have found that persons with disability, especially women, access and use health services as much as or more than persons without disability [1, 31]. Given the higher frequency of use of healthcare, studies argue that persons with disability may have more opportunities for engaging with the health care systems, and in the case of HIV, knowing their HIV status [18]. We suspect that because of a larger proportion of persons with disability living with HIV (11.8%) in our study consistent with national estimates [32] persons with disability, especially women may have received more attention in knowing their HIV-positive status than women without disability. This attention may have helped equalize persons with disability’s access to ART to that of persons without disability. The higher awareness of the HIV-positive status and equal access to ART between persons with and without disability were possible in part because the Tanzanian Government scaled up and decentralized ART services, offered free of charge to every person visiting a health facility, as a part of the standard of care [33]. Alongside these efforts, stakeholders led by the Tanzania Commission for AIDS, and the National AIDS Control Programme, advocated for improving the policy and legislation environment to facilitate disabled persons’ access to HIV services [4]. The National AIDS Strategic Framework and National Guidelines for Management of HIV and AIDS, key policy documents that identify and allocate national resources towards specific HIV programme areas and population groups recognized persons with disability as a priority population for focused differentiated HIV services [6, 34]. As a result of these efforts, women with disability, may have known their HIV-positive status as well as, or even better, than persons without disability.

Our study’s third result was that men with disability on ART had lower odds of having suppressed HIV viral load than men without disability on ART. The evidence shows that men had lower HIV prevalence, lower odds of awareness of their HIV-positive status, access to ART and suppressing their HIV viral load than women living with HIV [28, 21]. A study covering Eswatini, Lesotho, Malawi, Zambia, and Zimbabwe found that compared to women, men were 72% less likely to have suppressed viral load [28]. In Tanzania the THIS report showed that 41.5% of male adults 15 years or older had suppressed viral load compared to 57.2% of their female peers [21]. Regarding disability, in a study in Tanzania, care givers with mental or physical disability were 16% less likely to report adhering to HIV treatment [20]. None of these studies showed differences between men with and without disability in suppressing their HIV viral load. In our study, the evidence to support the differences between men with and without disability on ART in their HIV viral load suppression was not strong although the corresponding adjusted odds ratio was low and significant. The association between the female sex and disability interaction term and HIV viral load suppression was not significant suggesting the association between HIV viral suppression and disability status is unlikely to differ between men and women.

Our finding about the poor HIV viral load suppression among men with disability on ART is important. The inequalities for men with disability in suppressing their HIV viral load is due to structural, political and cultural shortcomings, including a lack of accessibility, leading to exclusion and unequal opportunities [35]. This result supports the need for disability-inclusive HIV interventions to increase the awareness of HIV-positive status and HIV viral load suppression among men living with HIV in general and men with disability in particular. Examples of such interventions include HIV testing in the communities and homes, self-testing, implementing HIV testing and care incentives, shifting gender norms and practices to increase men’s access of HIV services, and promoting male-friendly health services [36, 37]. Men with disability may require support to address the deprivation, social exclusion, stigma and discrimination, ridicule, and verbal threats of violence they may face from men without disabilities and other members of the community [38]. These factors may prevent men with disability from accessing HIV services and suppressing their HIV viral load. Support towards men with disability, for example men unable to see or walk, could include increase access to suitable and functioning assistive devices to enhance their independent mobility and participation in community activities, and enhance personal capability to provide for oneself and family [38].

This study has a few limitations. We did not control for the quality of the health care services the respondents received which tends to be lower among persons with disability [1, 31] and can reduce their continued engagement in HIV care. However, a review of the inclusion of persons with disability in the health financing system in Tanzania conducted in 2013 found that most persons with disabilities reported being satisfied with the quality of services received [39]. Further, we did not control for the evolution of disability among PLHIV and its interaction with HIV prevalence, awareness of HIV-positive status, access to ART and HIV viral load suppression. The HIV infection can cause physical, sensory, and cognitive difficulties, potentially worsening them among persons with disability who contract HIV, and older PLHIV [5]. Effective HIV treatment has increased the lifespan of PLHIV. The net impact of HIV treatment on the onset and progression of disabilities among PLHIV is not known. Besides, we did not include people with albinism in our analyses because the WG-SS does not identify people with albinism in surveys using internationally comparable questions [23]. In Tanzania, and several other countries of east and southern Africa, people with albinism face the risk of violence and death because of superstitious beliefs that they are not human, and their body parts are a portent concoction for witchcraft [40]. Furthermore, beliefs that HIV can be cured by having sex with persons with albinism are common and may increase their vulnerability to HIV infection and prevent them from accessing HIV services [41]. The WG-SS has limited efficiency in detecting psychosocial issues and does not measure all disabilities [22]. In general, persons with disability, especially women, face sexual and gender-based violence, which may impact their access to HIV services [14, 11]. We did not control for violence in this study because the violence data set was not available at the time of this study. The observations for persons with disabilities were small leading to large confidence intervals for example for differences in viral suppression between men with and without disabilities. However, we used survey weights to calculate appropriate estimates at the population level. We used the THIS data, the only data set among the PHIA data sets that had HIV testing and treatment cascade and disability variables. Analyzing data sets for different countries containing the HIV testing and treatment cascade and disability variables may have yielded different results. Surveys that sample more persons with disability, include data on HIV biomarkers and HIV incidence, are required to better estimate differences in HIV prevalence and care cascade between persons with and without disability, to inform disability-inclusive HIV services [5].

## Conclusions

We found no differences in the odds of having HIV and access to ART between persons with and without disability in Tanzania. While PLHIV with disability were often aware of their HIV-positive status, men living with HIV with disability were disadvantaged in suppressing their HIV viral load, compared to their non-disabled counterparts. These differences are correctable with disability-inclusive programming. The interventions designed to meet the 95—95—95 target should account for inequalities related to age, residence, education, and wealth to reduce the HIV prevalence among persons with disability and enhance their access to HIV services. HIV surveys around the world should include questions on disability to measure potential differences in HIV prevalence and in attaining the 2025 HIV care cascade target between persons with and out disability.

### Electronic supplementary material

Below is the link to the electronic supplementary material.

**Supplementary Table A1**: The odds of having HIV, awareness of HIV-positive status, access to ART and HIV viral load suppression among persons with and without disability (unadjusted and adjusted Odds ratios, 95% confidence intervals, sample size) (THIS 2016 - 2017). **Supplementary Table B1**: The odds of having HIV, awareness of HIV-positive status, access to ART and HIV viral load suppression among persons with and without disability, females (unadjusted and adjusted Odds ratios, 95% confidence intervals, sample size) (THIS 2016 - 2017). **Supplementary Table C1**. The odds of having HIV, awareness of HIV-positive status, access to ART and HIV viral load suppression among persons with and without disability, males (unadjusted and adjusted Odds ratios, 95% confidence intervals, sample size) (THIS 2016 - 2017). **Supplementary Table 2**. Distribution of difficulties by type and degree, percent, 95% Confidence Interval, sample size (N=1831)

## Data Availability

All data generated or analysed during this study are available from the corresponding author on request, or can be requested at https://phia-data.icap.columbia.edu.

## Abbreviations

aOR: Adjusted odds ratios
ART: Antiretroviral therapy
CI: Confidence Interval
CRPD: Convention on the Rights of Persons with Disabilities
PHIA: Population-based HIV Impact Assessment Survey
PLHIV: People living with HIV
THIS: Tanzania HIV Impact Survey
UN: United Nations
WG-SS: Washington Group Short Set Questions
