# Supplementary material for: Differences between persons with and without disability in HIV prevalence, testing, treatment, and care cascade in Tanzania: a cross-sectional study using population-based data
Source: BMC Public Health. 2023 Oct 25;23:2096. doi: 10.1186/s12889-023-17013-8 (PMC10601322; doi:10.1186/s12889-023-17013-8)
Supplement: Supplementary file 1 — Supplementary Table A1: The odds of having HIV, awareness of HIV-positive status, access to ART and HIV viral load suppression among persons with and without disability (unadjusted and adjusted Odds ratios, 95% confidence intervals, sample size) (THIS 2016 - 2017). Supplementary Table B1: The odds of having HIV, awareness of HIV-positive status, access to ART and HIV viral load suppression among persons with and without disability, females (unadjusted and adjusted Odds ratios, 95% confidence intervals, sample size) (THIS 2016 - 2017). Supplementary Table C1. The odds of having HIV, awareness of HIV-positive status, access to ART and HIV viral load suppression among persons with and without disability, males (unadjusted and adjusted Odds ratios, 95% confidence intervals, sample size) (THIS 2016 - 2017). Supplementary Table 2. Distribution of difficulties by type and degree, percent, 95% Confidence Interval, sample size (N=1831) [file 12889_2023_17013_MOESM1_ESM.docx]

Supplementary Tables

| **Supplementary Table A1: The odds of having HIV, awareness of HIV-positive status, access to ART and HIV viral load suppression among persons with and without disability (unadjusted and adjusted Odds ratios, 95% confidence intervals, sample size) (THIS 2016 - 2017)** | | | | | | | | | | | |
| --- | --- | --- | --- | --- | --- | --- | --- | --- | --- | --- | --- |
| **Variable** | **HIV status** | |  | **Awareness of HIV-positive status** | |  | **Accessing ART** | |  | **HIV viral Load suppression** | |
|  | **Unadjusted** | **Adjusted** |  | **Unadjusted** | **Adjusted** |  | **Unadjusted** | **Adjusted** |  | **Unadjusted** | **adjusted** |
| **Disability status** (Ref: Not disabled) |  |  |  |  |  |  |  |  |  |  |  |
| **Disabled** | 1.35 (1.13 - 1.60) | 1.03 (0.86 - 1.23) |  | 1.99 (1.28 - 3.09) | 1.69 (1.06 - 2.68) |  | 2.84 (0.77 - 10.41) | 1.56 (0.45 - 5.46) |  | 0.69 (0.38 - 1.25) | 0.55 (0.26 - 1.18) |
| **Gender** (Ref:male) |  |  |  |  |  |  |  |  |  |  |  |
| Female | 1.92 (1.69 - 2.17) | 1.85 (1.63 - 2.09) |  | 1.69 (1.30 - 2.20) | 1.86 (1.40 - 2.45) |  | 2.36 (1.39 - 4.01) | 3.18 (1.82 - 5.53) |  | 1.57 (0.96 - 2.57) | 1.77 (1.02 - 3.09) |
| **Age** (Ref: 15-24) |  |  |  |  |  |  |  |  |  |  |  |
| 25-34 | 3.83 (3.04 - 4.83) | 3.64 (2.87 - 4.59) |  | 1.03 (0.65 - 1.64) | 1.10 (0.67 - 1.81) |  | 0.60 (0.20 - 1.82) | 0.68 (0.23 - 2.06) |  | 1.49 (0.61 - 3.68) | 1.55 (0.65 - 3.70) |
| 35-44 | 7.18 (5.70 - 9.04) | 6.54 (5.18 - 8.25) |  | 1.83 (1.15 - 2.91) | 2.02 (1.22 - 3.32) |  | 1.13 (0.37 - 3.46) | 1.50 (0.48 - 4.69) |  | 1.27 (0.54 - 3.00) | 1.57 (0.62 - 3.98) |
| 45-54 | 7.06 (5.64 - 8.83) | 6.43 (5.10 - 8.11) |  | 2.44 (1.57 - 3.79) | 2.62(1.59 - 4.30) |  | 2.51 (0.78 - 8.04) | 3.38 (0.97 - 11.83) |  | 1.26 (0.51 - 3.13) | 1.70 (0.65 - 4.49) |
| 55+ | 3.66 (2.83 - 4.73) | 3.21 (2.42 - 4.25) |  | 1.58 (0.91 - 2.75) | 1.67 (0.91 - 3.05) |  | 4.21 (0.49 - 36.08) | 5.19 (0.67 - 40.34) |  | 1.75 (0.54 - 5.64) | 2.83 (0.67 - 11.92) |
| **Location (Ref: rural)** | |  |  |  |  |  |  |  |  |  |  |
| Urban | 1.47 (1.26 - 1.71) | 1.87 (1.51 - 2.31) |  | 1.32 (1.00 - 1.74) | 1.17 (0.80 - 1.71) |  | 1.01 (0.57 - 1.79) | 0.79 (0.36 - 1.73) |  | 1.04 (0.64 - 1.69) | 1.10 (0.65 - 1.86) |
| **Education level** (Ref: no education) |  |  |  |  |  |  |  |  |  |  |  |
| Primary | 0.89 (0.75 - 1.06) | 0.97 (0.80 - 1.17) |  | 1.43 (1.04 - 1.96) | 1.37 (0.97 - 1.95) |  | 1.16 (0.52 - 2.60) | 1.23 (0.56 - 2.70) |  | 0.90 (0.41 - 1.97) | 0.96 (0.47 - 1.99) |
| Secondary and higher | 0.42 (0.33 - 0.52) | 0.66 (0.50 - 0.87) |  | 1.13 (0.69 - 1.84) | 1.36 (0.80 - 2.31) |  | 1.17 (0.39 - 3.54) | 1.39 (0.52 - 3.72) |  | 0.94 (0.39 - 2.28) | 1.21 (0.49 - 2.99) |
| **Wealth Quintiles** (Ref: Q1) |  |  |  |  |  |  |  |  |  |  |  |
| Q2 | 1.06 (0.82 - 1.37) | 1.04 (0.81 - 1.35) |  | 1.40 (0.90 - 2.17) | 1.30 (0.81 - 2.08) |  | 0.64 (0.25 - 1.64) | 0.69 (0.27 - 1.75) |  | 1.91 (0.69 - 5.28) | 2.10 (0.78 - 5.65) |
| Q3 | 1.45 (1.12 - 1.89) | 1.32 (0.99 - 1.75) |  | 1.76 (1.16 - 2.68) | 1.61 (1.02 - 2.56) |  | 1.07 (0.41 - 2.77) | 1.05 (0.41 - 2.65) |  | 1.13 (0.49 - 2.60) | 1.12 (0.47 - 2.68) |
| Q4 | 1.47 (1.15 - 1.86) | 1.10 (0.83 - 1.46) |  | 1.57 (1.00 - 2.45) | 1.23 (0.67 - 2.253) |  | 0.58 (0.24 - 1.43) | 0.58 (0.20 - 1.70) |  | 1.40 (.58 - 3.36) | 1.27 (0.52 - 3.09) |
| Q5 | 1.01 (0.78 - 1.32) | 0.73 (0.51 - 1.04) |  | 1.75 (1.09 - 2.81) | 1.35 (0.71 - 2.57) |  | 1.70 (0.49 - 5.93) | 1.53 (0.38 - 6.18) |  | 1.03 (0.38 - 2.78) | 0.87 (0.29 - 2.59) |
| N | 31579 | 31552 |  | 1782 | 1780 |  | 1101 | 1100 |  | 1,026 | 1025 |
| 19 (0.06%; 19/33004) and 8 (0.02%; 8/33004) observations were missing in education and wealth. 45 (2,8% of 1619) observations were missing in access to ART for people with no disability and 4 (1,9% of 212) observation for people with disability. HIV prevalence was based on respondents 15 years and older (n=32977); PLHIV 15 years and older (n=1831); and all other variables on adult (15 years and older) PLHIV without disability (n=1619) and with disability (n=212). | | | | | | | | | | | |

| **Supplementary Table B1: The odds of having HIV, awareness of HIV-positive status, access to ART and HIV viral load suppression among persons with and without disability, females (unadjusted and adjusted Odds ratios, 95% confidence intervals, sample size) (THIS 2016 - 2017)** | | | | | | | | | | | |
| --- | --- | --- | --- | --- | --- | --- | --- | --- | --- | --- | --- |
| **Variable** | **HIV prevalence** | |  | **Awareness of HIV-positive status** | |  | **Accessing ART** | |  | **HIV viral Load suppression** | |
|  | **Unadjusted** | **Adjusted** |  | **Unadjusted** | **Adjusted** |  | **Unadjusted** | **Adjusted** |  | **Unadjusted** | **adjusted** |
| **Disability status** (Ref: Not disabled) |  |  |  |  |  |  |  |  |  |  |  |
| **Disabled** | 1.27 (1.05 - 1.55) | 1.06 (0.86 - 1.31) |  | 2.21 (1.20 - 4.05) | 1.93 (0.99 - 3.76) |  | 4.27 (0.97 - 18.73) | 2.08 (0.41 - 10.62) |  | 0.72 (0.33 - 1.58) | 0.71 (0.29 - 1.72) |
| **Age** (Ref: 15-24) |  |  |  |  |  |  |  |  |  |  |  |
| 25-34 | 3.47 (2.72 - 4.43) | 3.24 (2.54 - 4.13) |  | 1.20 (0.73 - 1.99) | 1.23 (0.73 - 2.05) |  | 0.68 (0.18 - 2.53) | 0.78 (0.21 - 2.88) |  | 1.02 (0.40 - 2.62) | 0.92 (0.33 - 2.56) |
| 35-44 | 5.88 (4.52 - 7.65) | 5.25 (4.07 - 6.78) |  | 1.94 (1.18 - 3.16) | 1.90 (1.17 - 3.11) |  | 1.34 (0.38 - 4.74) | 1.36 (0.39 - 4.72) |  | 1.31 (0.52 - 3.32) | 1.24 (0.42 - 3.66) |
| 45-54 | 5.61 (4.35 - 7.23) | 5.02 (3.84 - 6.55) |  | 2.54 (1.45 - 4.47) | 2.49 (1.37 - 4.53) |  | 4.76 (1.00 - 22.61) | 4.79 (0.91 - 25.05) |  | 0.63 (0.24 - 1.67) | 0.63 (0.21 - 1.90) |
| 55+ | 2.69 (2.01 - 3.60) | 2.40 (1.72 - 3.34) |  | 1.27 (0.70 - 2.29) | 1.27 (0.68 - 2.36) |  | 34.85 (5.21 - 233.03) | 38.45 (5.40 - 273.97) |  | 0.98 (0.22 - 4.42) | 1.00 (0.20 - 5.04) |
| **Location (Ref: rural)** |  |  |  |  |  |  |  |  |  |  |  |
| Urban | 1.72 (1.44 - 2.06) | 2.09 (1.63 - 2.67) |  | 1.36 (1.00 - 1.86) | 1.47 (0.97 - 2.22) |  | 0.59 (0.29 - 1.22) | 1.03 (0.40 - 2.66) |  | 1.07 (0.57 - 2.00) | 1.28 (0.70 - 2.32) |
| **Education level** (Ref: no education) |  |  |  |  |  |  |  |  |  |  |  |
| Primary | 1.09 (0.89 - 1.34) | 1.02 (0.81 - 1.29) |  | 1.54 (1.06 - 2.25) | 1.48 (1.01 - 2.18) |  | 1.24 (0.51 - 2.97) | 2.22 (0.88 - 5.59) |  | 0.88 (0.32 - 2.46) | 0.87 (0.38 - 2.01) |
| Secondary and higher | 0.50 (0.38 - 0.66) | 0.62 (0.44 - 0.88) |  | 1.35 (0.71 - 2.55) | 1.62 (0.81 - 3.22) |  | 0.77 (0.22 - 2.64) | 1.87 (0.53 - 6.51) |  | 0.77 (0.26 - 2.27) | 0.76 (0.29 - 2.02) |
| **Wealth Quintiles** (Ref: Q1) |  |  |  |  |  |  |  |  |  |  |  |
| Q2 | 1.01 (0.76 - 1.36) | 0.98 (0.73 - 1.32) |  | 1.29 (0.74 - 2.27) | 1.11 (0.63 - 1.94) |  | 1.12 (0.30 - 4.14) | 1.21 (0.32 - 4.57) |  | 5.49 (1.44 - 20.92) | 5.40 (1.42 - 20.53) |
| Q3 | 1.49 (1.09 - 2.03) | 1.29 (0.93 - 1.80) |  | 2.03 (1.23 - 3.36) | 1.63 (0.93 - 2.82) |  | 0.54 (0.16 - 1.85) | 0.51 (0.15 - 1.78) |  | 1.31 (0.46 - 3.74) | 1.23 (0.43 - 3.51) |
| Q4 | 1.66 (1.25 - 2.21) | 1.15 (0.82 - 1.59) |  | 1.48 (0.87 - 2.51) | 1.00 (0.52 - 1.96) |  | 0.20 (0.06 - 0.63) | 0.19 (0.05 - 0.73) |  | 2.05 (0.70 - 6.00) | 1.83 (0.66 - 5.05) |
| Q5 | 1.21 (0.91 - 1.60) | 0.82 (0.55 - 1.20) |  | 1.58 (0.93 - 2.68) | 1.01 (0.52 - 1.97) |  | 0.50 (0.12 - 2.09) | 0.46 (0.09 - 2.36) |  | 1.04 (0.31 - 3.55) | 0.89 (0.27 - 2.94) |
| N | 17829 | 17812 |  | 1230 | 1228 |  | 804 | 803 |  | 759 | 758 |

| **Supplementary Table C1. The odds of having HIV, awareness of HIV-positive status, access to ART and HIV viral load suppression among persons with and without disability, males (unadjusted and adjusted Odds ratios, 95% confidence intervals, sample size) (THIS 2016 - 2017)** | | | | | | | | | | | |
| --- | --- | --- | --- | --- | --- | --- | --- | --- | --- | --- | --- |
| **Variable** | **HIV status** | |  | **Awareness of HIV-positive status** | |  | **Accessing ART** | |  | **HIV viral Load suppression** | |
|  | **Unadjusted** | **Adjusted** |  | **Unadjusted** | **Adjusted** |  | **Unadjusted** | **Adjusted** |  | **Unadjusted** | **adjusted** |
| **Disability status** (Ref: Not disabled) |  |  |  |  |  |  |  |  |  |  |  |
| **Disabled** | 1.43 (1.03 - 2.00) | 0.98 (0.70 - 1.38) |  | 1.74 (0.82 - 3.68) | 1.30 (0.58 - 2.91) |  | 2.08 (0.23 - 19.08) | 1.43 (0.16 - 12.59) |  | 0.63 (0.22 - 1.79) | 0.23 (0.06 - 0.81) |
|  |  |  |  |  |  |  |  |  |  |  |  |
| **Age** (Ref: 15-24) |  |  |  |  |  |  |  |  |  |  |  |
| 25-34 | 4.98 (2.82 - 8.79) | 4.95 (2.80 - 8.75) |  | 0.85 (0.27 - 2.67) | 0.91 (0.27 - 3.08) |  | 0.51 (0.08 - 3.35) | 0.64 (0.07 - 6.14) |  | 4.73 (0.59 - 38.06) | 5.98 (0.43 - 83.17) |
| 35-44 | 11.60 (6.85 - 19.65) | 11.15 (6.56 - 18.95) |  | 2.33 (0.73 - 7.48) | 2.38 (0.67 - 8.52) |  | 1.34 (0.19 - 9.42) | 1.59 (0.14 - 17.60) |  | 2.77 (0.32 - 23.74) | 4.09 (0.27 - 63.08) |
| 45-54 | 11.95 (7.03 - 20.32) | 11.32 (6.67 - 19.24) |  | 3.26 (1.17 - 9.13) | 3.31 (1.08 - 10.14) |  | 2.40 (0.35 - 16.44) | 2.45 (0.23 - 26.64) |  | 10.26 (1.13 - 93.13) | 22.10 (1.47 - 332.52) |
| 55+ | 6.74 (3.82 - 11.89) | 6.19 (3.49 - 10.98) |  | 3.10 (0.98 - 9.82) | 3.13 (0.95 - 10.28) |  | 3.48 (0.23 - 52.04) | 3.98 (0.19 - 82.83) |  | 9.90 (1.04 - 94.53) | 33.63 (1.48 - 762.50) |
|  |  |  |  |  |  |  |  |  |  |  |  |
| **Location (Ref: rural)** |  |  |  |  |  |  |  |  |  |  |  |
| Urban | 1.02 (0.81 - 1.28) | 1.52 (1.15 - 2.01) |  | 1.02 (0.64 - 1.63) | 0.76 (0.43 - 1.33) |  | 1.40 (0.56 - 3.52) | 0.41 (0.11 - 1.54) |  | 0.82 (0.35 - 1.92) | 0.59 (0.19 - 1.84) |
|  |  |  |  |  |  |  |  |  |  |  |  |
| **Education level** (Ref: no education) |  |  |  |  |  |  |  |  |  |  |  |
| Primary | 0.74 (0.55 - 1.01) | 0.75 (0.55 - 1.03) |  | 1.36 (0.75 - 2.48) | 0.97 (0.50 - 1.90) |  | 1.27 (0.30 - 5.35) | 0.93 (0.20 - 4.43) |  | 1.05 (0.31 - 3.55) | 0.69 (0.18 - 2.60) |
| Secondary and higher | 0.38 (0.24 - 0.58) | 0.66 (0.41 - 1.08) |  | 1.03 (0.41 - 2.56) | 0.87 (0.32 - 2.35) |  | 2.77 (0.40 - 19.32) | 1.31 (0.16 - 10.57) |  | 1.58 (0.34 - 7.34) | 1.37 (0.24 - 7.82) |
|  |  |  |  |  |  |  |  |  |  |  |  |
| **Wealth Quintiles** (Ref: Q1) |  |  |  |  |  |  |  |  |  |  |  |
| Q2 | 1.14 (0.81 - 1.62) | 1.14 (0.80 - 1.62) |  | 1.62 (0.90 - 2.91) | 1.63 (0.85 - 3.14) |  | 0.64 (0.20 - 2.03) | 0.63 (0.19 - 2.10) |  | 0.70 (0.14 - 3.38) | 1.38 (0.22 - 8.53) |
| Q3 | 1.40 (1.01 - 1.93) | 1.38 (0.97 - 1.96) |  | 1.43 (0.79 - 2.61) | 1.65 (0.85 - 3.21) |  | 1.50 (0.42 - 5.37) | 1.87 (0.57 - 6.07) |  | 0.80 (0.17 - 3.91) | 1.25 (0.25 - 6.31) |
| Q4 | 1.12 (0.79 - 1.59) | 1.01 (0.67 - 1.52) |  | 1.52 (0.72 - 3.23) | 1.75 (0.71 - 4.34) |  | 1.60 (0.38 - 6.86) | 2.70 (0.42 - 17.42) |  | 0.62 (0.13 - 2.97) | 1.00 (0.16 - 6.21) |
| Q5 | 0.65 (0.39 - 1.08) | 0.54 (0.31 - 0.93) |  | 1.82 (0.68 - 4.89) | 2.50 (0.83 - 7.47) |  | 14.84 (5.33 - 41.29) | 21.01 (3.41 - 129.24) |  | 1.08 (0.19 - 6.24) | 1.67 (0.15 - 8.84) |
| N | 13750 | 13740 |  | 552 | 552 |  | 297 | 297 |  | 267 | 267 |

| Supplementary Table 2. Distribution of difficulties by type and degree, percent, 95% Confidence Interval, sample size (N=1831) | | | | | |
| --- | --- | --- | --- | --- | --- |
| Difficulty | No difficulty |  | Some difficulty |  | A lot of difficulty  /Unable to |
| Albino | 99.6 (99.0 - 99.8),1822 |  |  |  | 0.4 (0.1 - 1.0), 8 |
| Seeing | 93.4 (92.0 - 94.5), 1707 |  | 6,5 (5,4 - 7,9), 120 |  | 0.1 (0.0 - 0.3), 4 |
| Hearing | 97.7 (96.5 - 98.4), 1790 |  | 2.3 (1.5 - 3.4),39 |  | 0.1 (0.0 - 0.3), 2 |
| Walking | 96.3 (95.1 - 97.2), 1753 |  | 2.8 (2.1 - 3.7), 66 |  | 0.9 (0.4 - 2.0),12 |
| Communicating | 99.4 (98.9 - 99.7),1819 |  | 0.5 (0.2 - 1.1), 10 |  | 0.0 (0.0 - 0.3), 1 |
| Remembering | 98.7 (97.8 - 99.2), 1807 |  | 1.3 (0.8 - 2.2), 24 |  | 0 |
| Self-care | 99.2 (98.5 - 99.6), 1818 |  | 0.6 (0.3 - 1.2), 8 |  | 0.2 (0.1 - 0.6),5 |
